# The role of bacterial outer membrane vesicles in inflammatory response of acute-on-chronic liver failure

**DOI:** 10.3389/fmicb.2025.1608137

**Published:** 2025-07-04

**Authors:** Xiaojing Qin, Shuang Wang, Zhanyao Yan, Ninghui Zhao, Jia Yao

**Affiliations:** Third Hospital of Shanxi Medical University, Shanxi Bethune Hospital, Shanxi Academy of Medical Sciences, Tongji Shanxi Hospital, Taiyuan, China

**Keywords:** acute-on-chronic liver failure, bacteria, outer membrane vesicles, inflammation, cirrhosis, gut-liver axis

## Abstract

Acute-on-chronic liver failure (ACLF) is a clinical syndrome that manifests as acute deterioration of liver function due to a series of etiologies and triggers in patients with pre-existing chronic liver diseases. Systemic inflammatory response is the major feature of ACLF. Gut microbiota dysbiosis impairs the intestinal barrier, facilitating the translocation of microorganisms and their metabolites into the liver and thereby exacerbating liver inflammation and disease progression. Recent studies have revealed that bacterial outer membrane vesicles (OMVs) derived from gut microbiota act as key mediators in microbiota-host cell communication. This article elucidates the possible roles of OMVs in ACLF inflammation and their underlying mechanisms.

## 1 Introduction

Acute-on-chronic liver failure (ACLF) is a clinical syndrome that manifests as an acute decompensation of liver function in patients with pre-existing chronic liver disease, and is characterized by a high case-fatality rate and multiple organ failure (Clària et al., 2023; Br and Sarin, 2023). In ACLF patients, the rapid decline in liver function leads to impaired detoxification and waste disposal, which results in toxin overload and physiological dysfunction, with a series of associated clinical signs and symptoms. Cirrhosis is a common underlying condition of ACLF (Artru and McPhail, 2024) as it will progress to ACLF following an acute injury from sources such as bacterial/viral infection, alcoholism, drugs, or variceal bleeding (Wang et al., 2022; Br and Sarin, 2023; Clària et al., 2023). However, despite its diverse etiologies, ACLF has been typically characterized by a common systemic inflammatory response and immunosuppression (Br and Sarin, 2023).

The pathogenesis of ACLF remains unclear. The systemic inflammation hypothesis has gained widespread recognition, positing that ACLF is an acute flare of pre-existing systemic inflammation in patients with decompensated cirrhosis that can cause immune-mediated tissue damage (Clària et al., 2016; Arroyo et al., 2021; Samuel, 2021). Accordingly, inflammation is a pivotal factor throughout the course of ACLF. The inflammation extends beyond the liver in ACLF, with the gastrointestinal (GI) tract contributing significantly to its pathogenesis (Trebicka et al., 2021a; Kim et al., 2021; Hsu and Schnabl, 2023). The extant literature suggests that gut microbiota dysbiosis is prevalent in patients suffering from cirrhosis and liver failure when compared with healthy populations. The interaction between gut-derived bacteria and their products with the liver is initiated through the gut-liver axis and the intestinal mucosal barrier, which results in the induction of liver inflammation and fibrosis (Trebicka et al., 2021a; Hsu and Schnabl, 2023). In addition, systemic inflammation in ACLF patients has been found to be linked to intestinal flora dysbiosis and altered metabolic pathways (Kim et al., 2021; Moreau et al., 2020).

Recent research has demonstrated an increased interest in the role of outer membrane vesicles (OMVs) released by gut bacteria in disease processes (Chen et al., 2023b; Liang et al., 2022; Liu et al., 2021b; Xie et al., 2023b). OMVs play key roles in mediating gut bacteria-host interactions and can be involved in inflammatory responses and immune regulation (Liang et al., 2022; Han et al., 2024). Gut microbes and their byproducts activate liver inflammation and drive the progression of liver diseases (Hsu and Schnabl, 2023). Gut microbiota-derived OMVs exacerbate liver inflammation and fibrosis (Zahmatkesh et al., 2022; Natsui et al., 2023; Dorner et al., 2024). Therefore, these OMVs may trigger and worsen the progression from cirrhosis and other chronic liver diseases to ACLF, which may inform future intervention strategies.

## 2 Inflammation in ACLF and its etiologies

**Figure 1:**
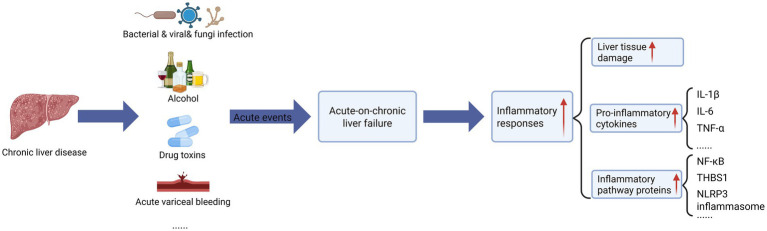
The effect of acute event strikes on acute-on-chronic liver failure (ACLF). On the basis of chronic liver disease, acute liver failure triggered by acute injury events (including bacterial/viral/fungi infection, alcohol abuse, drug/toxicity poisoning, acute vascular rupture and bleeding, etc.) leads to uncontrolled systemic inflammatory response, which is manifested by (1) hepatic tissue injury (hepatocellular necrosis/apoptosis), (2) pro-inflammatory cytokine storm (significant elevation of IL-1β, IL-6, and TNF-α, etc.), (3) activation of inflammatory pathway proteins (NF-κB signaling, upregulation of THBS1 expression, and elevation of NLRP3 inflammasome), which synergistically form a vicious cycle of “inflammation-injury.” The figure was created with BioRender.com (www.biorender.com).

ACLF has a variety of etiologies and triggers including bacteria, viruses, alcohol, and toxins, which elicit systemic inflammation that is pivotal to disease progression (Figure 1) (Br and Sarin, 2023). These factors induce hepatocyte necrosis, releaseing of inflammatory mediators and cytokines that amplify liver inflammation, creating a positive feedback loop enhancing liver injury. Additionally, overactivated hepatic immune cells secrete excess inflammatory mediators and cytokines, disrupting immunomodulation and exacerbating liver damage (Albillos et al., 2022; Martin-Mateos et al., 2019). These effects commonly feature inflammatory processes. Studies in mouse models of liver disease (nonalcoholic steatohepatitis (MASH), liver injury, ACLF, etc.) confirmed that inflammation itself can lead to hepatic fibrosis and liver injury and accelerate disease progression (Jiang et al., 2018; Zhang et al., 2023; Cheng et al., 2024; Lv et al., 2024). In the blood and liver tissues of individuals with cirrhosis and ACLF, pro-inflammatory cytokines such as interleukin (IL)-1β, IL-6, and tumor necrosis factor (TNF-*α*) (Clària et al., 2016; Tilg et al., 1992), as well as important inflammatory pathway proteins, such as nuclear factor- κB (NF-κB) (Zhang et al., 2022b), and thrombospondin-1 (THBS1) (Hassan et al., 2024), and NOD-like receptor protein 3 (NLRP3) inflammasome (Zhang et al., 2024b) are elevated. In essence, all the etiologies and triggers of ACLF have a shared feature in that they promote inflammation.

### 2.1 Pathogen infection

Infection, caused by either bacterial, viral, and fungi pathogens, is the most common etiology and trigger of chronic liver disease in ACLF (Mezzano et al., 2022; Xu et al., 2024; Yang et al., 2025). A multicenter ACLF cohort study (Fernández et al., 2018) revealed that 37% of patients had bacterial infection upon admission, and 46% of others developed infection within 4 weeks, indicating prevalent bacterial infections in ACLF patients, along with an even higher mortality rate among patients with bacterial infection-induced ACLF (Fernández et al., 2018). It has been demonstrated that patients suffering from cirrhosis are susceptible to bacterial infections and are more likely to progress to ACLF than the general population (Wong et al., 2021; Arroyo et al., 2021; Piano et al., 2024). *Helicobacter pylori*, a common gastric pathogen, is linked to liver disease (Li et al., 2023; Wang et al., 2021a; Mohammadi et al., 2023; Wang et al., 2023), with studies showing its infection correlates with inflammatory markers in cirrhotic patients as *H. pylori* infection has been observed to stimulate the release of pro-inflammatory cytokines. *H. pylori* eradication improves inflammatory marker and vascular mediator levels and lowers the incidence of cirrhosis complications (Abdel-Razik et al., 2020; Li et al., 2023). A single-center prospective cohort study by Abdel-Razik et al. also showed that *H. pylori* infection promotes the development of decompensated cirrhosis such as hepatic decompensation, ascites, deterioration of coagulation indices, and the development of esophagogastric varices, (Abdel-Razik et al., 2020) and induces ACLF progression. *Escherichia coli* and *Klebsiella pneumoniae* are also frequently observed in ACLF patients with bacterial infections (Wong et al., 2021; Zhang et al., 2022a). *E. coli* promotes the progression of cirrhosis and the development of complications, including hepatic encephalopathy by activating immune cells and promoting the release of inflammatory factors (Natsui et al., 2023; Wu et al., 2021b). Viral infections, particularly hepatitis B virus (HBV) and hepatitis E virus, are key triggers of ACLF (Zhu et al., 2022; Wang et al., 2022; Wu et al., 2021a), leading to rapid liver function decline via viral replication, immune dysregulation, immune cell exhaustion, and cytokine storms (Li et al., 2022a; Wang et al., 2022; Zhu et al., 2022). Table 1 lists the major pathogens that trigger ACLF and the substances or mechanisms by which they may act.

**Table 1:** Major pathogens of acute-on-chronic liver failure (ACLF).

References	Pathogens		Substances or mechanisms of action
Chang et al. (2021) and Wang et al. (2022)	Virus	HBV	Double-stranded DNA viral genes, viral replication, high viral load, hepatocyte damage; immune damage and depletion
Shin and Jeong (2018) and Kanda et al. (2020)		HAV	Single-stranded RNA viral genes, viral replication, hepatocyte damage, impact on interferon response, and activation of immune response
Panda and Varma (2013), Buti et al. (2025), and Kumar et al. (2008)		HEV	Single-stranded RNA viral genes, viral replication, hepatocyte damage, and activation of immune responses
Al-Aalim et al. (2021), Shen et al. (2023), and Dorner et al. (2024)	Bacteria	*Escherichia coli*	Endotoxins (LPS), SCFAs, flagellins, OMVs
Okushin et al. (2018), Waluga et al. (2015), Zahmatkesh et al. (2022), and Mohammadi et al. (2023)		*Helicobacter pylori*	Endotoxin (LPS), cytotoxin-associated gene A (CagA), vacuolating cytotoxin A (VacA), OMVs
Wang et al. (2020), Li et al. (2014), and Li et al. (2025)		*Klebsiella pneumoniae*	Endotoxin (LPS), bacterial hairs, iron carriers, OMVs
Surewaard et al. (2018) and De Kimpe et al. (1995)		*Staphylococcus aureus*	Peptidoglycan, lipophosphatidic acid, α-toxin (AT)
Hartmann and Schnabl (2023) and Verma et al. (2023)	Fungi	Candida	β-glucan, mannan, Candida lysin, Candida leucine aminopeptidase
Hartmann and Schnabl (2023)		Aspergillus	β-glucan, mannan, aflatoxin

### 2.2 Alcohol

Alcohol overdose is a significant risk factor for ACLF. Long-term or addicted alcohol use is linked to a higher incidence of ACLF (Singal and Shah, 2019). Long-term alcohol use alters gut microbiota composition, disrupts the integrity and permeability of the intestinal mucosal barrier, and facilitates the translocation of pathogen-associated molecular pattern molecules (PAMPs, including bacteria). When the PAMPs enter the liver and the circulation, they act on liver macrophages through lipopolysaccharide (LPS) to induce inflammation, resulting in a series of pathological changes in the liver (Szabo, 2015; Parlesak et al., 2000). Additionally, ethanol metabolism generates reactive oxygen species, damaging mitochondria and inducing apoptosis (Zhou et al., 2001; Lu et al., 2008), thus contributing to liver failure.

### 2.3 Drug toxins

Drug-induced liver injury is also linked to ACLF progression, beyond chronic liver disease (Devarbhavi et al., 2019). Some drugs can elicit immune system hyperreactivity and oxidative stress, damaging liver cell structure and functions (Qian et al., 2024; Ye et al., 2018). The metabolism of some drugs produces toxic products. For example, acetaminophen (APAP) produces excess N-acetyl-p-benzoquinone imine (NAPQI), which depletes glutathione (GSH), leading to mitochondrial dysfunction, increased oxidative stress, and direct toxicity to hepatocytes, resulting in hepatocellular necrosis (Ye et al., 2018; Li et al., 2022b). In addition, the combination of environmental factors and individual differences can contribute to drug-induced damage, potentially causing ACLF (Yu et al., 2017).

### 2.4 Acute variceal hemorrhage

Acute variceal hemorrhage is a precipitating factor in liver failure (Wang et al., 2022). Acute variceal hemorrhage leads to liver ischemia, which compromises the integrity of the intestinal barrier. This increases the risk of bacterial infections due to increased bacterial translocation. This can induce a pro-inflammatory or exacerbated inflammatory response (Sarin et al., 2019; Triantos et al., 2022).

Inflammation is a pivotal factor in the clinical course and outcome of ACLF. Chronic liver disease is marked by persistent inflammation and fibrosis progression (Hammerich and Tacke, 2023). In a prospective study (Trebicka et al., 2021b), 65% of ACLF patients enrolled had evident triggers of systemic inflammation. Notably, patients with two or more precipitating factors had higher levels of inflammatory markers (including white blood cell count, neutrophil count, and monocyte count, and C-reactive protein) than in patients with no clinically obvious precipitating factors or a single precipitating factor (Trebicka et al., 2021b). Bacterial infections, alcohol overdose, and viral activity can lead to the release of PAMPs, which triggers inflammation, impairs organ function, and thus induces ACLF. Engelmann et al. also reviewed the core mechanisms of acute decompensation in cirrhosis and the pathophysiology associated with its progression to ACLF, stating that after the occurrence of various triggering factors leading to acute decompensation, a cascade of pathogenetic processes will follow (Engelmann et al., 2021). In short, excessive release of inflammatory mediators can disrupt the “pro-inflammatory and anti-inflammatory” balance and exacerbates liver damage, potentially leading to decompensation (Engelmann et al., 2021). These studies reveal that inflammation both sustains disease and triggers ACLF.

## 3 Microbiota in ACLF

In cirrhosis and ACLF patients, the imbalanced intestinal microbiota is characterized by reduced probiotics and increased opportunistic pathogens (e.g., Enterococcus and Bacteroides). The latter can produce toxins, trigger inflammation, and aggravate liver damage (Kim et al., 2021; Solé et al., 2021). Cirrhosis and ACLF patients often exhibit compromised intestinal barrier, allowing endotoxins and bacteria to translocate into the liver, causing infections and systemic inflammation, and worsening liver damage and decompensation (Trebicka et al., 2021c; Kim et al., 2021). The role of gut flora in cirrhosis and ACLF has been widely recognized, and therefore modulating gut flora has also been considered as a potential therapeutic tool (Sharma et al., 2022; Patel et al., 2022). And studies have shown modulating gut microbiota through microbiota transplantation or the use of antibiotics can help to treat or alleviate symptoms and inflammation in cirrhosis or liver failure patients (Sharma et al., 2022, Patel et al., 2022).

### 3.1 Altered microbiome in ACLF

A study performed over 10 years ago found that increased intestinal bacterial translocation was linked to the development of ACLF, attributing it to the gut-liver axis dysfunction (Verbeke et al., 2011). Increasing studies have characterized gut microbiota alterations in cirrhosis and ACLF (Chen et al., 2011; Bajaj et al., 2014; Qin et al., 2014; Bajaj et al., 2019; Tranah et al., 2021; Bajaj et al., 2012; Chen et al., 2015; Mehtani et al., 2021; Wang et al., 2021b; Yao et al., 2021). The gut microbiome in cirrhosis demonstrates reduced diversity with pathogenic dominance and loss of beneficial taxa. This leads to compromised integrity of the intestinal barrier and increased intestinal permeability. These alterations facilitate the translocation of bacteria and their metabolites (Sharma et al., 2024; Chen et al., 2011; Bajaj et al., 2014; Qin et al., 2014; Bajaj et al., 2019; Tranah et al., 2021), thereby fostering the development and progression of ACLF. Further exploration of the gut microbiome in ACLF revealed reduced abundance of protective anti-inflammatory bacteria (e.g., Bacteroides, Ruminococcae, and Trichalium) and increased abundance of harmful bacteria (e.g., Pasteurella and streptococci) (Chen et al., 2015; Bajaj et al., 2014), which correlated with disease severity and mortality (Chen et al., 2015; Yao et al., 2021; Solé et al., 2021).

Microbiome alteration is an active participant and influencing factor, rather than a passive factor, in disease progression (Sharma et al., 2024; Bajaj et al., 2014; Solé et al., 2021). Relevant studies in cirrhotic mice, mice with liver injury, and other animal models of liver disease have clearly revealed the significant role of microbiota in multiple key pathological processes including intestinal barrier integrity, gut permeability, systemic inflammation, neuroinflammation, and blood–brain barrier integrity (Kang et al., 2024; Liu et al., 2020; Fouts et al., 2012), which jointly contribute to host homeostasis and health. A decline in barrier-maintaining, immunomodulatory, and anti-inflammatory bacteria may enhance the host’s vulnerability to pro-inflammatory damage triggered by harmful gut bacteria, pathogens, or environmental toxins. Such a microecological imbalance worsens the original pathology and can even initiate or advance new disease processes (Chen et al., 2011; Bajaj et al., 2014; Chen et al., 2015).

Microbiome changes are present early in the development of chronic liver disease (Schnabl and Brenner, 2014), which indicates that the intestinal microbiota has been damaged in the prodromal state of ACLF, reinforcing microbial dysbiosis as a functional contributor to ACLF. Recent literature has indicated the close association between intestinal dysbiosis and liver diseases (Bajaj et al., 2019; Stols-Gonçalves et al., 2023; Trebicka et al., 2021a; Hsu and Schnabl, 2023; Solé et al., 2021; Sharma et al., 2022). Studies on the subject of gut microbiota in patients diagnosed with cirrhosis or viral hepatitis revealed signs of microflora dysbiosis in these patients, and the alterations in the composition of their gut microbiota were associated with progression of ACLF (Wang et al., 2021b; Solé et al., 2021). Thus, microbiota dysbiosis is an early marker of ACLF, and microbiome monitoring and intervention in the early stages of ACLF can be valuable for the prevention, timely detection, and effective management of ACLF.

In addition to stool-based gut microbiome changes, small intestinal bacterial overgrowth (SIBO) has also been recognized as another common form of gut dysbiosis in patients diagnosed with cirrhosis and other liver diseases (Pande et al., 2009; Alexiou et al., 2024; Gudan et al., 2022). SIBO impairs nutrient absorption, further taxing compromised liver function (Gudan et al., 2023; Adike and DiBaise, 2018). Furthermore, SIBO fosters gut inflammation and compromises barrier integrity, thus facilitating harmful bacteria and their metabolites entry into the circulation, which exacerbate liver damage and trigger immune responses and systemic inflammation (Lauritano et al., 2010; Pande et al., 2009; Alexiou et al., 2024), finally exacerbating ACLF via the vicious circle of the gut-liver axis. In fact, the cirrhosis-SIBO connection underscores the pivotal function of gut microbiota in advancing liver disease.

Microbiota transplantation studies affirmed the crucial function of gut microbiota in ACLF (Jiang et al., 2024; Zhang et al., 2024c; Bajaj et al., 2018; Sharma et al., 2022). Gut microbiota transplantation can rebalance microbial communities in cirrhotic patients, boosting the diversity of gut bacteria by increasing the relative abundance of potentially beneficial flora (e.g., Bifidobacterium) while reducing the release of pro-inflammatory cytokines (e.g., IL-1β, IL-6). Also, it can help to alleviate inflammation, liver injury, and complications such as ACLF and hepatic encephalopathy in cirrhotic patients (Bajaj et al., 2018, Sharma et al., 2022). In a study on gut microbiota transplantation in alcohol-related ACLF patients, transplantation significantly improved short- to medium-term survival and reduced inflammatory markers (e.g., IL-1β, IL-6) compared to controls (Sharma et al., 2022). And the treatment group also showed more pronounced improvement in hepatic encephalopathy and ascites, as well as a lower incidence of adverse events, such as gastric hemorrhage (Sharma et al., 2022). In addition, an animal-level research has shown that gut microbiota transplantation significantly lowered aspartate transaminase and alanine transaminase levels, mitigated hepatocyte necrosis, enhanced intestinal barrier function, reduced inflammation, and enhanced disease regression (Zhang et al., 2024c; Wang et al., 2017). Zhao et al. (2021) found that administering cirrhotic mice with intragastric bacterial suspension from cirrhosis patients exacerbated intestinal barrier damage and liver injury, accelerating cirrhosis progression (compared to bacterial suspension from healthy individuals). Therefore, the gut microbiome in certain cirrhotic patients may harbor factors that can drive ACLF progression.

### 3.2 ACLF inflammation and the microbiota-gut-liver/-brain axis

As the gut microbiome significantly impacts human health and disease (Fan and Pedersen, 2021), the gut-liver axis has gained prominence in liver disease research (Albillos et al., 2020; Tilg et al., 2022; Hsu and Schnabl, 2023; Schnabl and Brenner, 2014). The gut-liver axis denotes the intricate network linking the intestine and the liver via anatomic structures, circulation, neural control, and metabolism pathways for bidirectional interactions of the gut and its microbiota with the liver (Hsu and Schnabl, 2023; Albillos et al., 2020). This network plays a crucial role in maintaining homeostasis, particularly in regulating metabolism and immune responses (Hsu and Schnabl, 2023, Albillos et al., 2020). The liver is the first organ affected by gut bacteria and their products after the entry of these pathogens into the intestinal barrier and can be influenced by the gut microbiome in multiple ways (Hsu and Schnabl, 2023; Trebicka et al., 2021a). The significance of microbiota in liver disease, in relation to the translocation of gut bacteria and their byproducts, has increasingly been recognized; however, the mechanism behind gut diversity changes driving disease progression remains unclear.

Most gut bacteria subtly interact with host cells under a healthy gut status. They are confined to the gut lumen and separated from epithelial cells by the mucosal barrier, thus preventing bacterial invasion (Hiippala et al., 2018; Turner, 2009). As a result, their communication with host cells (e.g., epithelial and immune cells) relies on the release of a variety of secretory factors including OMVs. These secretory factors, acting as messengers, convey bacterial information across the mucosal barrier and interact with host cells to influence their physiology (Luo et al., 2021; Dorner et al., 2024; Liang et al., 2022), potentially significantly contributing to ACLF pathology.

Bacteria and their products (e.g., LPS, bacterial DNA, and peptidoglycan) can trigger the immune response (Gasaly et al., 2021), inflammation, and hepatocyte apoptosis, ultimately accelerating liver failure (Won et al., 2021). In the setting of ACLF, the translocation of gut bacteria and their metabolites (e.g., the translocation of LPS into the liver and systemic circulation) modulates the immune response and inflammation in the liver (Hasa et al., 2022; Suriguga et al., 2022; Chen et al., 2021). As a key component of Gram-negative bacteria outer membrane, LPS is also an effective trigger of immune responses. Its blood level is markedly elevated in cirrhotic patients (Simbrunner et al., 2023; Lin et al., 1995). And LPS can be recognized by pattern recognition receptors such as Toll-like receptors (TLRs), which are located on the cell surface of the liver, activate TGF-β and NF-κB signaling pathways, induce strong inflammatory responses and fibrosis, and regulate immune function (Fan et al., 2021; Engelmann et al., 2020). Increased expression of hepatic TLR4 and circulating TLR4 ligands in cirrhotic patients heightens cellular sensitivity to endotoxins generated by translocated Gram-negative bacteria, which is associated with disease development and prognosis (Engelmann et al., 2020). The TLR4 pathway is pivotal in ACLF development, with the recognized LPS triggering intracellular signaling and inflammatory cytokine release (Engelmann et al., 2020). A recent prospective study performed macrogenomic sequencing of fecal samples from patients with cirrhosis, and analysis showed that microbiomic changes significantly impact ACLF incidence (Solé et al., 2021), reinforcing the importance of gut-liver axis in the progression and prognosis of cirrhosis. Furthermore, studies have shown that 40–50% of ACLF patients have unexplained severe systemic inflammation, and it is assumed that gut bacterial translocation and resultant “pathogens” and their metabolites may play pivotal roles (Moreau et al., 2013). All of the above reveal the importance of gut bacteria and their bacterial products in the progression and prognosis of liver disease through the enterohepatic axis.

Studies reveal a bidirectional gut-liver-brain axis communication (Mayer et al., 2015; Liu et al., 2021a), with healthy homeostasis of the axis preventing harmful intestinal contents from reaching the brain via two barriers: the homeostasis of intestinal permeability and the normal liver function (Liu et al., 2021a). Gut microbes can exert direct or indirect effects on neurons to control the production and/or release of neurotransmitters by sending signals to the brain through neurons, endocrine, and immune mediators (Cani and Knauf, 2016). Mounting evidence suggests that changes in the structure of the gut microbiota and the effects of its metabolites, along with local or systemic inflammation, impaired liver metabolism, and leaky gut, can promote the occurrence of liver failure and hepatic encephalopathy (Liu et al., 2021a; Trebicka et al., 2021c; Sharma et al., 2024). Cirrhosis involves systemic inflammation and endotoxin spread, triggering neuroinflammation and causing neurological complications (e.g., cognitive impairment) (Butterworth, 2019). Interestingly, it has been found that *H. pylori*-derived OMVs can cross the GI, blood–brain, and blood-CSF barriers, reaching the brain to induce peripheral and central inflammation, potentially triggering Alzheimer’s disease (Xie et al., 2023a). As *H. pylori* is a common bacterium in the GI tract, it is assumed that *H. pylori* OMVs may also promote the occurrence of hepatic encephalopathy.

**Figure 2:**
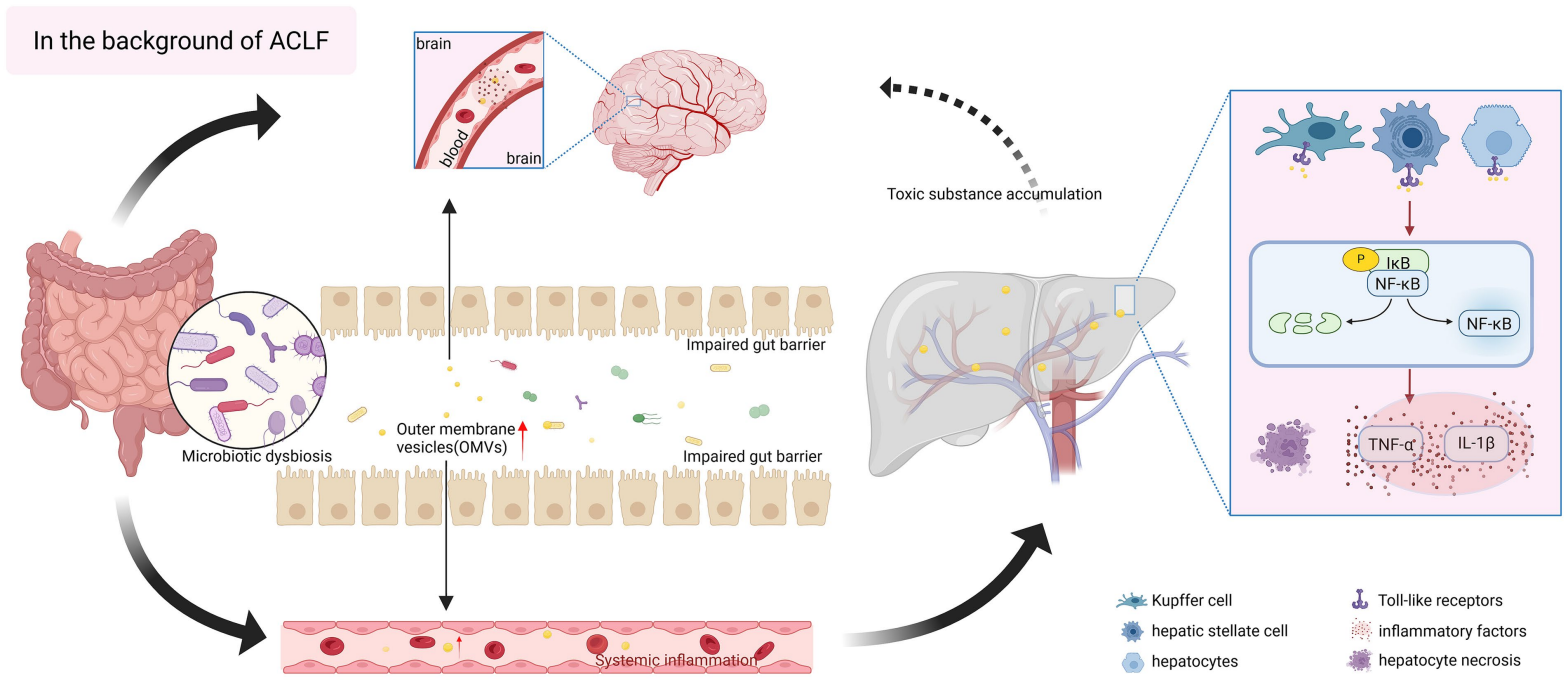
Hypothesized pro-inflammatory role of bacterial outer membrane vesicles (OMVs) in acute-on-chronic liver failure (ACLF). In the context of ACLF, the composition of gut microorganisms is disordered, and intestinal bacteria release a large number of OMVs, which are rich in pathogen-associated molecular patterns (PAMPs), and at the same time, there is damage to the gut barrier, and intestinal permeability is enhanced, which is more conducive for OMVs to cross the gastrointestinal barrier, and to reach liver tissues through the gut-liver axis, and they can act on a wide variety of cells in the liver, and they can act on hepatocytes directly, inducing OMVs can act on various cells in the liver, directly affecting hepatocytes, inducing oxidative stress and apoptosis, and exacerbating liver injury; they can also act on Kupffer cell and hepatic stellate cells, and bind to Toll-like receptors on the surface of the cells, activating inflammatory signaling pathways, such as NF-κB, and releasing a large number of pro-inflammatory cytokines (IL-1β, TNF-α), exacerbating hepatic fibrosis, and triggering systemic inflammatory responses. The inflammation within the liver mediated by OMVs can further spread to induce multi-organ failure. In addition, OMVs can also cross the blood–brain barrier, invade the brain and activate neuroinflammation, while the severely impaired liver detoxification function promotes the accumulation of ammonia and other toxic substances, affecting the nervous system, a series of chain reactions, which lay the groundwork for the occurrence of hepatic encephalopathy. The figure was created with BioRender.com (www.biorender.com).

It is generally believed that, in the context of ACLF, bacteria-secreted factors may penetrate the intestinal barrier, thus initiating an immune response cascade that likely drives disease progression. Notably, Heetanshi *et al.* (Jain et al., 2024) isolated LPS-positive extracellular vesicles from portal vein plasma and liver tissues, confirming the presence of OMVs in liver. Accordingly, we propose that OMVs play an equally pivotal role in ACLF. As the main secretions and communication pathways of bacteria, OMVs have the ability to cross biological barriers such as the intestinal barrier and the blood–brain barrier to deliver “cargo,” although their specific mechanisms in ACLF remains understudied. We have created a schematic diagram to illustrate this assumption (Figure 2).

## 4 Bacterial OMVs

OMVs were first reported in the 1960s, with researchers observing spherical vesicles from *E. coli* under electron microscopy, initially believed to be apoptotic fragments (Bishop and Work, 1965). These vesicles were later termed outer membrane vesicles (OMVs). Bacteria extracellular vesicles (bEVs) are a general term for nanoscale vesicles with a lipid bilayer membrane structure secreted by bacteria, and encompasses a variety of types of vesicles, such as cytoplasmic membrane vesicles (CMVs), OMVs, and so on (Ho et al., 2024). Among them, OMVs are extracellular vesicles derived from Gram-negative bacteria. OMVs, 20–300 nm in size (Avila-Calderón et al., 2021), are vesicle-like structures that are shed from the bacterial cells surface and released into the extracellular area bacteria during the growth of Gram-negative, and are primarily composed of parental proteins, lipids, and nucleic acids (Avila-Calderón et al., 2021; Toyofuku et al., 2019; Toyofuku et al., 2023). For the purpose of this review, we focus on OMVs, the extracellular membrane vesicles released by Gram-negative bacteria. Of course, we also recognize the potential relevance of extracellular vesicles (CMVs) released by Gram-positive bacteria in disease [see review for details (Xu et al., 2023, Sangiorgio et al., 2024)].

OMVs are produced by Gram-negative bacteria through a typical model of outer membrane vesiculation. There are multiple mechanisms by which Gram-negative bacteria shed OMVs, including a decrease in the number of lipoproteins attached to the peptidoglycan layer leading to outer membrane bulging, peptidoglycan residues with autolysin leading to outer membrane protrusion, and the effect of the charge carried by the LPS (Avila-Calderón et al., 2021). The secretion of OMVs is impacted by multiple factors, including bacterial growth stage, surrounding environmental conditions, cell physiological state, antibiotic exposure, and microbial interactions (Toyofuku et al., 2019). Most OMVs carry specific “cargo” molecules, conferring various functions such as virulence factor transmission, enhancing bacterial survival and pathogenicity, signal transduction, promoting interbacterial communication, and mediating bacteria-host information exchange (Jan, 2017; Sartorio et al., 2021). Compared with bacteria themselves, OMVs are more likely to cross tight junctions and biological barriers. As the “long-range weapons” for bacteria, they attack host tissues, facilitate bacterial colonization, impair host cell function, and compromise host defenses, thus playing key roles in the pathogenic processes (Toyofuku et al., 2023). OMVs can spread to distant tissues and are found in blood (Tulkens et al., 2020; Luo et al., 2021), gastric mucosa (Luo et al., 2021), liver (Jain et al., 2024), brain (Xie et al., 2023a), and feces (Park et al., 2018).

### 4.1 Roles of OMVs in immunomodulation

Gut microbiota-derived OMVs modulate the host’s immunity in ACLF. The OMVs elicit local and systemic pro-inflammatory immune responses by engaging innate and adaptive immune cells including macrophages, dendritic cells, and neutrophils (Tiku and Tan, 2021; Spari et al., 2024). OMVs are abundant in PAMPs, including DNA, RNA, lipoproteins, LPS, and toxins, which can bind to pattern recognition receptors located on the surface of the host’s immune cells, leading to complex mechanisms of immune activation (Liang et al., 2022; Liu et al., 2021b; Xie et al., 2023b) and triggering a cascade of biological responses. For instance, Yang et al. treated mouse bone marrow-derived macrophages (BMDMs) with the OMVs secreted by *Salmonella typhimurium*, in which promoted flagellin act as a stimulus for the activation of NLRC4 inflammatory vesicles, mediating caspase-1 activation and IL-1β secretion (Yang et al., 2020). Additionally, an investigation into the functions of OMVs from another flagellated bacterial pathogen, *Pseudomonas aeruginosa*, revealed the similar outcomes. Robust caspase-1 activation and IL-1β secretion were also observed (Yang et al., 2020). Davis et al. found that the OMVs derived from pathogenic *E. coli* contain a bacterial toxin known as cytotoxic necrotizing factor 1, which negatively affects the phagocytic and chemotactic capabilities of neutrophils (Davis et al., 2006). *Salmonella typhimurium* OMVs have been observed to induce an increase in the expression of CD86 and MHC class II molecules in dendritic cells, and to promote the production of pro-inflammatory mediators TNF-*α* and IL-12 (Alaniz et al., 2007). Furthermore, the OMVs have been shown to possess antigenic properties that mediate the immune response of protective B cells and T cells (Alaniz et al., 2007). It has also been demonstrated that intraperitoneal injection of OMVs from LPS- and outer membrane protein-rich *E. coli* or fecal bacterial extracellular vesicles containing Gram-negative and Gram-positive bacterial EVs induced sepsis-like systemic inflammation in mice (Park et al., 2010; Park et al., 2018). Subsequent research has further elucidated that *E. coli* OMVs can induce the release of macrophage inflammatory mediators in both *in vitro* and *in vivo* experimental models, and there are components other than LPS in *E. coli* OMVs mediating the pro-inflammatory effects (Svennerholm et al., 2017).

The OMVs are replete with LPS (Kaparakis-Liaskos and Ferrero, 2015), and they possess the capacity to deliver LPS to a variety of cell types including macrophages, neutrophils, and natural killer cells. Through interaction with the Toll-like receptor 4 (TLR4)/myeloid differentiation protein 2 (MD2)/CD14 receptor complex on the cell surface, LPS activates the NF-κB signaling pathway (Ciesielska et al., 2021). This activation triggers the secretion of pro-inflammatory cytokines (e.g., TNF-α and IL-1β) and enhances the production of free radicals, including nitric oxide and superoxide, ultimately exacerbating hepatic inflammation (Ciesielska et al., 2021). Furthermore, OMVs, as a vector for the intracellular transport of LPS, can activate caspases and the NLRP3 inflammasomes, further potentiating the inflammatory response (Vanaja et al., 2016). Several studies have confirmed the pro-inflammatory signaling pathway of OMVs described above (Zhang et al., 2024a; Engevik et al., 2021; Chen et al., 2023a; Gong et al., 2022). LPS-containing *fusobacterium nucleatum* OMVs have been shown to induce intestinal inflammation through the activation of TLR4 and subsequent downstream targets NF-κB and extracellular signal-regulated kinase (ERK) (Engevik et al., 2021). In a rat model of periodontitiss, the addition of the OMVs resulted in an upregulation of dendritic cell, T cell, and macrophage markers (Zhang et al., 2024a). Furthermore, it was observed that the secretion of inflammatory factors was promoted by the triggering of NF-κB signaling pathway, the activation of NLRP3 inflammasomes, and the exertion of pathogenicity (Zhang et al., 2024a). The diabetic rat model demonstrated that OMVs can be transported into the renal tubular mesenchyme through the disrupted intestinal vascular barrier (Chen et al., 2023a). In this model, abundant LPS has been shown to target renal tubular epithelial cells, inducing tubular mesenchymal inflammation and renal injury through the caspases-11 pathway (Chen et al., 2023a). *In vitro* experimentation with colorectal cancer cells treated with adherent invasive *E. coli* OMVs resulted in the up-regulation of TLR2/TLR4 expression, the down-regulation of cell junction-associated proteins, and the induction of inflammatory factor release (Nadalian et al., 2024). Notably, OMVs emitted by *E. coli* have the capacity to elicit mitochondrial dysfunction, mitochondria-mediated apoptosis, and inflammatory activation (Deo et al., 2020), which may be implicated in the pathogenesis of ACLF given that mitochondrial impairment is also a hallmark of ACLF (Moreau et al., 2020).

The heightened abundance of pro-inflammatory Gram-negative bacteria and the enhanced production of LPS in ACLF (Cesaro et al., 2011; Clària et al., 2016; Wu et al., 2022; Pan et al., 2012) suggest that the gut microbiome in ACLF may be a significant source of LPS-rich OMVs. Elevated levels of LPS have been detected in the blood of ACLF patients. However, the methodologies employed in these studies did not differentiate between cell-free, monomeric LPS and OMVs-contained LPS. The LPS detected in the blood of ACLF patients may be OMV-associated.

OMVs engage with host cells through the binding of ligands to pattern recognition receptors (PRRs; including TLRs) located on the cell surface or to intracellular receptors such as nucleotide-binding oligomerization domain (NOD) proteins (Thay et al., 2014; Marion et al., 2019). In the pathological trajectory of ACLF, the predominant PRR families involved in the recognition of PAMPs and damage-associated molecular patterns (DAMPs) are TLRs and NOD-like receptors (NLRs) (Zhang et al., 2021; Engelmann et al., 2020). Alterations in TLR2 and TLR4 within the TLR family are associated with the pathological processes of ACLF. As a key component of the TLR family, TLR2 plays a central role in detecting bacterial cell wall components, lipoproteins, and other PAMPs from both Gram-positive and Gram-negative bacteria. Research has evidenced that TLR2 expression was upregulated in the immune cells of patients with hepatitis B virus-related ACLF (HBV-ACLF) (Xu et al., 2017), facilitating the secretion of inflammatory cytokines. Moreover, the enhanced expression of TLR2 was positively associated with the degree of liver injury and the severity of the disease in ACLF patients (Xu et al., 2017). The activation of TLR4 facilitates the recruitment of inflammatory cells and regulates the expression of inflammatory signaling pathways (Seki et al., 2007). Evidence indicates that TLR4 signaling is a crucial determinant in the pathogenesis of ACLF (Engelmann et al., 2020). TLR4 mediates LPS-induced tissue damage and potentiates cellular sensitivity to LPS derived from Gram-negative bacteria (Engelmann et al., 2020). In rodent models of ACLF, treatment with TAK-242, a TLR4 antagonist, has been demonstrated to mitigate LPS-induced hepatocyte apoptosis, decrease cytokine production in hepatocytes and monocytes, lower circulating IL-1β levels, and ameliorate systemic inflammation (Engelmann et al., 2020; Engelmann et al., 2022). NLR proteins, including the NLRP3 inflammasome complex which recognizes a spectrum of MAMPs and DAMPs, have been implicated in the pathogenesis of ACLF (Li and Jiang, 2021; Zhang et al., 2024b; Taru et al., 2024). In a prospective controlled study, Li et al. found elevated levels of NLRP3 in both serum and peripheral blood mononuclear cells of patients with ACLF, and increased expression of the related signaling pathway molecules procaspase-1 and pro-1β, which were positively correlated with disease severity (Li and Jiang, 2021).

### 4.2 OMVs and intestinal epithelium

OMVs released from intestinal lumen can cross the intestinal mucosal barrier and adhere to epithelial cells (Liang et al., 2022; Liu et al., 2021b). Such an effect may be exacerbated in ACLF patients, as these patients frequently exhibit a compromised intestinal barrier function. The interactions of OMVs with epithelial cells can precipitate the secretion of cytokines, which regulates cellular proliferation and impacts the expression of tight junction proteins (Fiocca et al., 1999). Critically, studies have demonstrated that OMVs are capable of traversing the epithelial layer and accessing the lamina propria through either paracellular or transcellular routes, where they subsequently engage with resident immune cells (Krsek et al., 2023; Kaparakis-Liaskos and Ferrero, 2015). OMVs derived from diverse bacterial species, including *Campylobacter jejuni*, *H. pylori*, and *Fusobacterium nucleatum*, among others, are capable of down-regulating the expression of key tight junction proteins (Elmi et al., 2016; Turkina et al., 2015; Nie et al., 2022; Liu et al., 2021b). This downregulation directly contributes to attenuation of the epithelial barrier function and potentially facilitates the translocation of bacteria and their products from the intestinal lumen to the deeper intestinal layers (Elmi et al., 2016; Turkina et al., 2015; Nie et al., 2022; Liu et al., 2021b). Tulkens et al. (2020) discovered that individuals exhibiting compromised intestinal barrier functionality exhibited a markedly elevated concentration of bacterial extracellular vesicles within their plasma as compared to healthy controls. Furthermore, there was a robust positive correlation between the abundance of these vesicles and the increased plasma level of zonulin-1, a pivotal biomarker indicative of intestinal barrier dysfunction (Tulkens et al., 2020). In an *in vitro* model of simulated colitis, the concentration of bacterial extracellular vesicles detected on the basal side increased with the decrease in zonulin-1 level, which further confirmed the capability of OMVs to translocate via the paracellular route (Tulkens et al., 2020). OMVs in ACLF have the potential to downregulate the expression of critical tight junction proteins, thereby compromising the integrity of the epithelial barrier; as a result, bacterial byproducts can be translocated into the liver and other organs more easily, thereby amplifying the systemic inflammatory response.

### 4.3 OMVs and liver

OMVs can affect the functionality of diverse cell types, and their translocation beyond the intestinal lumen will precipitate enduring and extensive impacts. The diffuse spread of OMVs throughout the body enables gut-resident bacteria to exert indirect, long-distance effects (Juodeikis and Carding, 2022; Hendrix and De Wever, 2022; Tulkens et al., 2020). An investigation into OMVs biodistribution indicated that, following oral administration of labeled OMVs to mice, the *in vivo* distribution of OMVs was predominantly localized to the GI tract and liver, suggesting that OMVs are capable of reaching and accumulating in the liver via GI translocation in the host (Jones et al., 2020). Significantly, this experiment was conducted in healthy mice, implying that OMVs can traverse the intestinal barrier even without the presence of intestinal injury (Jones et al., 2020). Furthermore, Dorner et al. (2024) found that in both patients and murine models of primary sclerosing cholangitis (PSC) complicated by inflammatory bowel disease (IBD), OMVs secreted by intestinal commensal bacteria crossed the intestinal mucosal barrier and accumulated within the liver. By activating the TLR4 and NLRP3-GSDMD signaling pathways, these OMVs lead to the activation of hepatocytes and stellate cells, promoting liver inflammation and fibrosis.

Evidence suggests that individuals exhibiting compromised GI barriers exhibit elevated levels of circulating bacterial extracellular vesicles relative to healthy individuals (Tulkens et al., 2020). Although this finding has not been corroborated in patients with ACLF, enhanced intestinal permeability and reduced intestinal tight junction protein expression are frequently noted in individuals with ACLF (Assimakopoulos et al., 2012; Kim et al., 2021; Hasa et al., 2022). It is plausible to hypothesize that the concentration of circulating bacterial extracellular vesicles, including OMVs, may be increased in this patient population. These OMVs, which potentially harbor inflammatory genes, could contribute to exacerbation of the inflammatory response observed in ACLF.

Within the intestinal milieu, OMVs demonstrate a superior capacity for traversing biological barriers compared to intact bacterial cells, thereby facilitating their access to liver tissues with greater ease. Throughout their transit, OMVs have the potential to encapsulate and transport toxins, antigens, and bioactive compounds present on the bacterial surface. Upon reaching the liver, these vesicles can elicit a cascade of immune responses and induce pathological alterations. Initially, several studies have demonstrated that liver macrophages are capable of internalizing microbe-originated EVs (Luo et al., 2021). Notably, EVs isolated from the feces of mice subjected to a high-fat diet are enriched with bacterial DNA. This bacterial DNA has the potential to induce hepatocellular inflammation and to activate hepatic stellate cell fibrosis through the cGAS/STING pathway, indicating the hepatotropic impact of microbial DNA contained within intestinal bacteria-derived EVs (Luo et al., 2022; Luo et al., 2021). Intraperitoneal administration of *Porphyromonas gingivalis*-derived OMVs to mice results in the accumulation of OMVs within the murine liver. Within this context, gingival proteases contained within the OMVs may modulate inflammation in macrophages and the immune response, consequently compromising hepatocyte metabolic function (Seyama et al., 2020). As previously discussed, an association exists between *H. pylori* infection and the pathogenesis of ACLF. Among the virulence factors of *H. pylori*, OMVs are particularly significant (Zahmatkesh et al., 2022). Bolori et al. (2023) and Shegefti et al. (2023) co-cultured *H. pylori* OMVs with LX-2 cells to assess the expression of hepatic fibrosis markers (E-cadherin, vimentin, β-catenin, *α*-SMA, and Snail) and autophagy inhibition markers (PI3K, AKT, and mTOR). Their findings revealed that *H. pylori* OMVs promoted liver fibrosis, inhibited autophagy of hepatic stellate cells, and mediated liver injury.

Natsui et al. (2023) isolated hepatocytes from wild-type mice and cultured them on Petri dishes. In comparison to cultures treated with LPS, the expression of Clec4e, TLR2, TLR3, and TLR4 was markedly elevated in hepatocyte cultures following the addition of *E. coli* OMVs. Furthermore, *in vitro* experiments showed that OMVs elicited inflammatory responses mediated by macrophages, neutrophils, and hepatic stellate cells in a dose-dependent manner, which can result in hepatocyte injury. In the *in vivo* experiments conducted in cirrhotic mice, OMVs triggered the activation of hepatic macrophages, leading to increased liver inflammation; in addition, OMVs activated hepatic stellate cells, causing the progression of fibrosis (Natsui et al., 2023). The OMVs were also detected in the ascitic fluid of cirrhotic patients (Natsui et al., 2023). All these findings suggested that OMVs were involved in the pathogenetic mechanisms of cirrhosis. Therefore, within the context of cirrhosis, the presence of OMVs may serve to augment hepatic injury and hasten the progression towards ACLF.

### 4.4 Prospects for the clinical anti-inflammatory therapy of OMVs

In addition to being pro-inflammatory and pathogenic, OMVs are promising therapeutic targets because they lack the ability to replicate, unlike intact bacteria. However, they have a similar structural composition, a unique nanoscale structure, and good biocompatibility, safety, immunogenicity, and targeting capabilities for disease treatment. OMVs can mediate intercellular information transfer for disease treatment due to their good biocompatibility, safety, immunogenicity, and targeting ability.

A substantial body of research has demonstrated that probiotic OMVs carry certain specific components that can ameliorate inflammation. It has been demonstrated that mucinophilic Ackermannia EVs have the capacity to attenuate liver injury and hepatic fibrosis in mice with liver injury (Keshavarz Azizi Raftar et al., 2021; Raftar et al., 2022). Furthermore, these EVs have been shown to modulate inflammation by restoring the composition of the intestinal flora and the barrier function (Keshavarz Azizi Raftar et al., 2021). In addition, polysaccharide A (PSA) from the OMVs of *B. fragilis* has been demonstrated to promote the synthesis of the anti-inflammatory factor IL-10 by binding to the TLR2 receptor in dendritic cells (Shagaleeva et al., 2024). This binding has been shown to alleviate DSS-induced colitis and to modulate the intestinal flora (Shagaleeva et al., 2024). In addition, OMVs derived from *Escherichia coli* Nissle 1917 have been found to express a PD1 agonist, a property that has been demonstrated to reduce the release of inflammatory factors by suppressing immune overactivation and, consequently, to ameliorate colitis (Hu et al., 2025).

In certain conditions, pathogenic OMVs have the potential to be utilized as a therapeutic agent, thereby transforming “enemies” into “friends.” Pan et al. employed the adhesive chemotaxis of *Stenotrophomonas maltophilia* OMVs to colon tissues and ice chips to construct hybrid liposomes for targeted drug delivery to restore intestinal homeostasis by alleviating inflammation and modulating the dysregulated intestinal epithelial barrier, redox balance, and intestinal microbiota for the treatment of IBD (Pan et al., 2025). In addition, there is considerable potential for OMVs in the domain of vaccine development (Sartorio et al., 2021), given their ability to stimulate the production of high levels of antibodies. A number of OMVs have already been successfully implemented in clinical settings, including the group *B meningococcal* OMV vaccine and the *Neisseria meningitidis* OMV vaccine (Petousis-Harris et al., 2017; Gan et al., 2021). Research is underway to develop vaccines for *Klebsiella pneumoniae* OMVs, *Acinetobacter baumannii* OMVs, and *Bordetella pertussis* OMVs (Huang et al., 2019; Assoni et al., 2021; Pschunder et al., 2024).

Many challenges remain in the clinical application of OMVs. However, continued exploration of the pathophysiological mechanisms of OMVs in the body and their translation into effective treatments is expected to lead to the integration of key inflammatory modulators or pathogens associated with ACLF disease into OMVs. This will facilitate the development of novel vaccines that target ACLF disease, attenuate inflammation, and prevent disease progression in future clinical practice.

## 5 Conclusion

ACLF is a clinical syndrome characterized by an intense systemic inflammatory response and is associated with a complex etiology and pathogenesis. Previous studies have demonstrated that the gut microbiota dysbiosis and impaired barrier function in ACLF drives the translocation of bacteria and their by-products through the gut-liver axis. This process subsequently activates the systemic immune system and induces excessive inflammatory responses and hepatocellular injury.

In this review, we emphasize that OMVs released by gut bacteria act as a “long-range weapon” of bacteria, delivering active ingredients and exacerbating systemic inflammation through activation of TLR signaling pathway, NLRP3 inflammasome, and mitochondrial dysfunction, echoing the pro-inflammatory effects of bacterial metabolites in previous studies. This finding aligns with the pro-inflammatory effects of bacterial metabolites previously observed in studies.

The study focuses on the contribution of OMVs in ACLF inflammation in the context of the disease background of ACLF. It reveals the mechanisms by which OMVs contribute to liver injury, including the activation of macrophages, neutrophils, and hepatic stellate cells. These exacerbate the storm of inflammatory factors and promote hepatic fibrosis and metabolic dysfunction. It is imperative to elucidate the potential pivotal functions of OMVs in the progression of inflammation in ACLF. This review systematically constructs a theoretical framework for the relationship between OMVs and inflammation in ACLF, providing a novel perspective to interpret the precipitous decline in liver function and uncontrolled inflammation in ACLF patients.

Furthermore, OMVs have been demonstrated to enhance disease progression and treatment outcomes through mechanisms such as drug loading, vaccine development, and OMVs modulation. Additionally, studies have explored the use of OMVs in the suppression of hepatic and intestinal inflammation. In light of the transport and biological activity of OMVs, future studies must further analyze the role of OMVs in ACLF. This analysis will provide a theoretical basis for the subsequent development of therapeutic strategies targeting OMVs to improve the severity and progression of ACLF.
